# Single ethanol binge causes severe liver injury in mice fed Western diet

**DOI:** 10.1097/HC9.0000000000000174

**Published:** 2023-06-14

**Authors:** Yu-Te Yeh, Xiangdong Wu, Yinyan Ma, Zhekang Ying, Ling He, Bingzhong Xue, Hang Shi, Youngshim Choi, Liqing Yu

**Affiliations:** 1 Department of Medicine, Division of Endocrinology, Diabetes, and Nutrition, University of Maryland School of Medicine, Baltimore, Maryland, USA; 2 Department of Medicine, Division of Cardiology, University of Maryland School of Medicine, Baltimore Street, Maryland, USA; 3 Departments of Pediatrics and Pharmacology & Molecular Sciences, Johns Hopkins University School of Medicine, Baltimore, Maryland, USA; 4 Department of Biology, Georgia State University, Atlanta, Georgia, USA

## Abstract

Alcohol-associated liver disease (ALD) and NAFLD often coexist in Western societies that consume energy-rich and cholesterol-containing Western diets. Increased rates of ALD mortality in young people in these societies are likely attributable to binge drinking. It is largely unknown how alcohol binge causes liver damage in the setting of Western diets.

**Approach and Results:**

In this study, we showed that a single ethanol binge (5 g/kg body weight) induced severe liver injury as shown by marked increases in serum activities of the 2 aminotransferases AST and ALT in C57BL/6J mice that have been fed a Western diet for 3 weeks. The Western diet plus binge ethanol-fed mice also displayed severe lipid droplet deposition and high contents of triglycerides and cholesterol in the liver, which were associated with increased lipogenic and reduced fatty acid oxidative gene expression. These animals had the highest Cxcl1 mRNA expression and myeloperoxidase (MPO)-positive neutrophils in the liver. Their hepatic ROS and lipid peroxidation were the highest, but their hepatic levels of mitochondrial oxidative phosphorylation proteins remained largely unaltered. Hepatic levels of several ER stress markers, including mRNAs for CHOP, ERO1A, ERO1B, BIM, and BIP, as well as Xbp1 splicing and proteins for BIP/GRP78 and IRE-α were also the highest in these animals. Interestingly, Western diet feeding for 3 weeks or ethanol binge dramatically increased hepatic caspase 3 cleavage, and the combination of the 2 did not further increase it. Thus, we successfully established a murine model of acute liver injury by mimicking human diets and binge drinking.

**Conclusions:**

This simple Western diet plus single ethanol binge model recapitulates major hepatic phenotypes of ALD, including steatosis and steatohepatitis characterized by neutrophil infiltration, oxidative stress, and ER stress.

## INTRODUCTION

Alcohol-associated liver disease (ALD) and NAFLD or metabolic-associated fatty liver disease (MAFLD) are the 2 most prevalent chronic liver diseases worldwide.^1^ ALD and NAFLD display similar liver pathologies, including steatosis, steatohepatitis, and fibrosis, which may ultimately progress to cirrhosis and cancer.^2^ Currently, treatment options are very limited. Lifestyle changes and alcohol abstinence remain major strategies. Advanced stages of ALD and NAFLD often require liver transplantation. While overnutrition-induced obesity is a major risk factor for NAFLD, ALD is caused by heavy drinking. In Western and other developed societies, alcohol drinking often coexists with overnutrition, which may lead to co-occurrences of ALD and NAFLD. It is important to determine how alcohol drinking and overnutrition interact with each other in the pathogenesis of liver disease.

In the past, ALD research was predominantly focused on chronic alcohol intake because of a greater chance of developing liver pathologies as alcohol drinking goes longer. Increased attention has now been paid to binge drinking, which was defined by the National Institute of Alcohol Abuse and Alcoholism (NIAAA) as 5 drinks for men and 4 for women in about 2 hours.^3^ In the United States, liver cirrhosis-related mortality rose to ~ 10% from 1999 to 2016 in millennials aged 25–34 years, which was entirely attributable to ALD.^4^ One potential reason for this increase may be due to increased binge drinking in this age group.^5–8^ Short-term binge drinking has been shown to cause hepatic steatosis and acute liver injury and is recognized as a risk factor for advanced ALD.^3,9^

It was reported that people with obesity or overweight had a greater risk of developing liver diseases than those with healthy weight when the same amount of alcohol was consumed.^10–12^ The onset and progression of ALD, like NAFLD, may involve multiple hits.^13^ In the setting of overnutrition and associated NAFLD, the liver may be sensitized or primed to overreact to alcohol intake, causing more damage. Indeed, a single dose of ethanol was shown to induce more severe liver injury in mice fed a high-fat diet (HFD) than those fed a low-energy chow diet.^14^ However, in developed societies, normal diets are enriched with not only energy but also cholesterol. Diets of this type are often referred to as Western diet, which differ from HFDs commonly used in the animal research of obesity. In general, the amount of cholesterol is 10-fold higher in Western diets (~0.2%, w/w) than HFDs (~ 0.02%, w/w). Excessive accumulation of free cholesterol is known to be cytotoxic in the liver and other organs.^15^ It is in this dietary background that alcohol drinking occurs in Western societies. Therefore, alcohol and Western diets are frequently co-consumed. ALD studies mimicking this dietary condition are scarce, and there is a clear need to conduct this research.

To explore how alcohol drinking in the setting of Western diets affects the pathogenesis of liver diseases, in this study, we examined the interplay of three weeks of Western diet feeding plus a single ethanol binge in driving acute liver injury. We observed that a single ethanol binge causes greater steatosis, injury, neutrophil infiltration, oxidative stress, and endoplasmic reticulum (ER) stress in the liver of mice fed a Western diet than those on a chow diet. Our study highlights the critical role of dietary compositions and nutritional status in modulating liver responses to alcohol drinking. By mimicking human diets and binge drinking in developed societies, we established a simple animal model of acute liver injury, frequent occurrences of which may result in advanced ALD and NAFLD.

## MATERIAL AND METHODS

### Mice and diets

Eight-week-old male C57BL/6J wild-type mice (Stock #: 000664, The Jackson Laboratory) were fed *ad libitum* for 3 weeks a laboratory rodent chow diet (5010, LabDiet; protein, 29% kcal; fat, 13% kcal; carbohydrate, 58% kcal and 0.0268% cholesterol (w/w), a Western diet (D12079B, Research Diet: protein, 17% kcal; fat, 41% kcal; carbohydrate, 43% kcal; and 0.21% cholesterol (w/w), or an HFD (D12492, Research Diet: protein, 20% kcal; fat, 60% kcal; carbohydrate, 20% kcal; and 0.015% cholesterol (w/w). On the last day of diet feeding, each diet group of mice was administered by oral gavage 1 dose of 31.25% ethanol in water at 5 g/kg body weight (BW) or an isocaloric dextrin-maltose solution 9 hours before necropsy during the daytime cycle. All mice were housed in a specific pathogen-free animal facility at 22°C with a 12  h light-dark cycle with lights on from 7:00 to 19:00. During experiments, mice had free access to food and water. Necropsy was done after an overdose of isoflurane by inhalation. Blood samples were collected by cardiac puncture and centrifuged at 1,500*×g* for 10 min at 4°C to obtain sera. Liver tissues were harvested and snap-frozen in liquid nitrogen for further analysis. All study protocols were approved by the Institutional Animal Care and Use Committees (IACUC) at the University of Maryland, Baltimore.

### Body composition analysis

Body composition of mice was analyzed using EchoMRI-100H Body Composition Analyzer (EchoMRI, LLC.).

### Measurements of serum parameters and hepatic lipids

Serum concentrations of cholesterol (Wako Diagnostics), phospholipids (Wako), triglycerides (Cayman Chemical), total bile acids (Crystal Chem), and non-esterified fatty acids (NEFA) (Wako) were determined enzymatically according to manufacturers’ instructions. These enzymatic assays were also used for the analysis of hepatic contents of cholesterol, phospholipids, and triglycerides after the extraction of lipids from liver samples, as described.^16^ Serum activities of alanine transaminase (ALT) and aspartate transaminase (AST) were determined using the assay kits from Pointe Scientific according to the company’s instructions.

### RNA extraction and reverse transcription-quantitative real-time polymerase chain reaction (qPCR)

The qPCR analysis of liver total RNAs was done as described.^16^ All qPCR primer sequences are available in Supplemental Table 1, http://links.lww.com/HC9/A314.

### Western blot analysis

Liver tissue homogenates were prepared using 0.3% SDS in RIPA Buffer (Sigma-Aldrich) containing protease and phosphatase inhibitors (Thermo-Fisher Scientific). After centrifugation at 12,000×*g* for 15 minutes, the supernatant was collected. The protein concentration of each supernatant was determined by Lowry Protein Assay Kit (Thermo-Fisher Scientific). Overall, 40 μg of proteins in each homogenate was subjected to 8% or 10% SDS-PAGE and then transferred onto a PVDF membrane. After being blocked with 5% non-fat milk in Tris-Buffered Saline-Tween (TBST) (20 mM Tris-HCl, pH 7.4, 150 mM NaCl, and 0.1% Tween-20) for 1 h at room temperature, the membrane was incubated overnight at 4°C with a primary antibody against FASN (ab128870), CD36 (sc7309), ACC (CST3676) that detects all isoforms of ACC, ATGL (CST2138), CHOP (CST2895), BIP/GRP78 (CST3177), IREα (CST3294), caspase 3 (CST9662), cleaved caspase 3 (CST9661), or α-tubulin (CST3873). The membrane was then washed with TBST 3 times, followed by incubation with either a goat anti-rabbit or mouse IgG horseradish peroxidase (HP)-conjugated secondary antibody (Thermo-Fisher Scientific). Protein bands were visualized with an enhanced chemiluminescence kit (Thermo-Fisher Scientific). The intensity of each protein band was quantified by densitometry using ImageJ. The expression level of a specific protein in a sample was determined after normalization to an invariant internal control protein on the same membrane.

### Histological analysis

Liver tissues were fixed in 10% (v/v) neutral buffered formalin solution, embedded in paraffin, and cut at 8 μm. Tissue sections were then stained with hematoxylin and eosin to visualize histological changes. Intracellular lipid droplets were visualized under an Olympus light microscope after Oil Red O or LipidSpot (70065, Biotium) staining of frozen liver sections that were embedded in the Tissue-Tek Optimum Cutting Temperature (OCT) compound. At least 3 images per specimen were obtained for analysis.

### Immunofluorescence staining

Immunofluorescence staining was performed using frozen or paraffin-embedded tissues. Tissue sections were stained with an antibody against F4/80 (CST70076) or myeloperoxidase (MPO) (ab208670) in phosphate buffered saline (PBS) containing 1% BSA at 4°C overnight. The sections were then incubated at room temperature for 1 hour with donkey anti-rabbit Alexa Fluor 594 secondary antibody. Cell nuclei were stained, and the tissue sections were mounted with ProLong Gold antifade reagent with DAPI (CST8961). At least 3 images per specimen were obtained under an Olympus fluorescence microscope.

### Statistical analysis

Statistical analyses were performed with GraphPad Prism 7.0. All results are expressed as mean ± SEM. Comparisons of data sets between the 2 groups were evaluated using an unpaired Student *t*-test. A *p* value ≤0.05 was considered statistically significant.

## RESULTS

### Single ethanol binge causes severe liver injury in mice fed a Western diet

**FIGURE 1:**
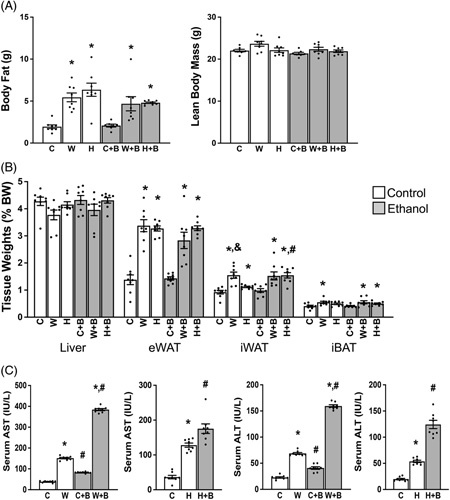
Western diet and single ethanol binge synergistically induce liver injury. Eight-week-old male mice were fed a chow or Western diet for 3 weeks, followed by a bolus of ethanol or maltose solution (as calorie control of ethanol) 9 hours prior to necropsy during the daytime cycle. (A) Body composition measured prior to necropsy by EchoMRI. (B) Liver and adipose tissue weights. (C) Serum activities of AST and ALT at necropsy. C, Chow diet; W, Western diet; H, High-fat diet. B, Ethanol binge.*p < 0.05 (Western diet or HFD vs. chow diet); ^#^ *p* < 0.05 (binge ethanol vs. maltose solution); ^&^ *p* < 0.05 (HFD vs. Western diet).

To determine how binge drinking, a situation that happens often in Western societies, affects liver health in the setting of Western diet consumption, we fed mice a Western diet or a control diet (chow diet or HFD) for 3 weeks, followed by ethanol binge as described under the methods. No mortality was observed. As expected, Western diet or HFD relative to chow diet feeding caused a significant increase in body fats without altering lean body mass (Figure 1A), and consistently, there were significant increases in adipose tissue weight while liver weight remained unaltered when normalized to BW (Figure 1B). Single ethanol binge had no impact on body composition and tissue-to-BW ratios regardless of diets (Figures 1A and B). There were no significant differences in fat mass, lean mass, and tissue-to-BW ratios between the Western diet and HFD groups except the inguinal subcutaneous white fat pad that was significantly heavier in the Western diet- than in HFD-fed animals. To determine whether ethanol binge causes greater liver injury in mice fed the Western diet than those on the chow diet or HFD, we measured serum activities of AST and ALT, the two aminotransferases that are often used clinically as surrogate markers for liver damage and function. Single ethanol binge raised serum AST and ALT activities modestly in chow- but markedly in Western diet-fed mice (Figure 1C). The absolute values of AST and ALT in the HFD group were higher and lower than those in chow and Western diet groups, respectively (Figure 1C). We did not present these data in the same bar figure due to measurements being done separately, though the same reagents and method were used. This observation suggests that Western diet plus ethanol binge synergistically induce liver injury that appeared to be more severe than that caused by HFD plus ethanol binge.

### Western diet plus single ethanol binge causes severe hepatic steatosis in mice

**FIGURE 2:**
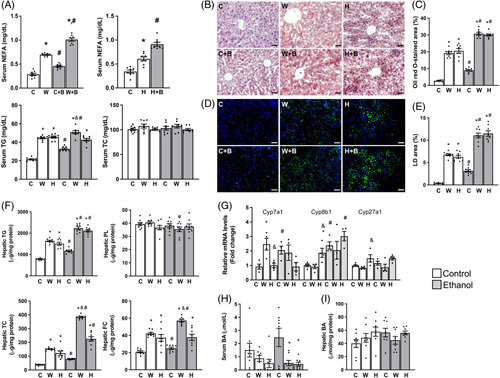
Western diet plus single ethanol binge synergistically increases hepatic steatosis. The same mice described under Figure 1 were used. (A) Serum levels of NEFA, TG, and total cholesterol (TC) at necropsy. (B–E) Oil red O (B and C) and LipidSpot (D and E) staining and quantification of hepatic lipid droplets. (F) Hepatic contents of TG, total cholesterol (TC), free cholesterol (FC), and phospholipids (PL). (G) Hepatic levels of mRNAs for key bile acid synthetic enzymes. (H) Serum concentrations of total bile acids (BA). (I) Hepatic contents of total bile acids (BA). *p < 0.05 (Western diet or HFD vs. chow diet); ^#^ *p* < 0.05 (binge ethanol vs. maltose solution); ^&^ *p* < 0.05 (HFD vs. Western diet). Abbreviations: C, Chow diet; W, Western diet; H, High-fat diet. B, Ethanol binge. eWAT, epididymal white adipose tissue; iWAT, inguinal subcutaneous white adipose tissue; iBAT, interscapular brown adipose tissue.

Western diets, HFD, and ethanol consumption each are known to alter lipid metabolism. Indeed, 3 weeks of Western diet or HFD feeding significantly raised serum concentrations of non-esterified fatty acids (NEFAs) and triglycerides (TGs), though not cholesterol (Figure 2A). Ethanol binge also raised serum NEFAs in mice regardless of diets, consistent with the established role of ethanol binge in stimulating adipose lipolysis.^17^ It is estimated that ~60% of fats deposited in the liver are derived from adipose lipolysis.^18^ Consistently, lipid droplet staining of liver sections by both Oil red O and LipidSpot dye revealed a substantial increase in lipid droplets in Western diet- and HFD-fed mice compared to chow-fed mice, with the highest increase seen in ethanol binge-treated mice consuming Western diet or HFD (Figures 2B-E). Similar changes were observed in hepatic contents of triglycerides, though not phospholipids (Figure 2F). Although both the Western diet and HFD increased hepatic contents of total and free cholesterol and ethanol binge increased total cholesterol regardless of diets, the Western diet plus ethanol binge group showed the highest increase in hepatic contents of total and free cholesterol with ethanol binge failed to increase free cholesterol in HFD-fed mice. These findings demonstrate that the Western diet plus ethanol binge or HFD plus ethanol binge causes severe hepatic steatosis, but only the former induces hepatic deposition of cytotoxic free cholesterol. A major pathway for the liver to get rid of free cholesterol is to convert it to bile acids by several key enzymes, such as cytochrome P450 family 7 subfamily A member 1 (Cyp7A1), cytochrome P450 family 8 subfamily B member 1 (Cyp8B1), and cytochrome P450 family 27 subfamily A member 1 (Cyp27A1). Changes in hepatic mRNAs for these proteins appeared to be diet dependent and so did their responses to ethanol binge (Figure 2G). Three weeks of Western diet or HFD relative to chow diet feeding lowered serum levels of bile acids, but this lowering became significant only after the ethanol binge (Figure 2H). No differences were observed in the hepatic content of total bile acids among all groups (Figure 2I).

### Single ethanol binge increases lipogenic and suppresses fatty acid oxidative gene expression in the liver

**FIGURE 3:**
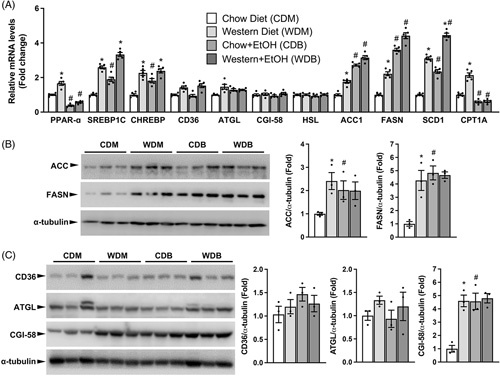
Single ethanol binge increases lipogenic and reduces fatty acid oxidative gene expression in the liver. The same mice described under Figure 1 were used. (A) Relative hepatic levels of mRNAs for genes critically implicated in fatty acid oxidation (Ppara and Cpt1a), fatty acid synthesis (Srebp1c, Chrebp, Acc1, Fasn, and Scd1), fatty acid transport (Cd36), and intracellular lipid droplet lipolysis (Atgl, Hsl, and Cgi-58). (B and C) Western blots and densitometry analysis of the lipogenic proteins ACC and FASN (B), the fatty acid transporter CD36, and the lipolytic proteins ATGL and CGI-58 (C) in the liver. *p < 0.05 (Western diet vs. chow diet); ^#^ *p* < 0.05 (binge ethanol vs. maltose solution).

HFD has been used in ALD research. The goal of this study was to establish an animal model of ALD by mimicking human diets and binge drinking behavior. Subsequent studies were, therefore, focused on the Western diet when compared to a regular rodent diet. Hepatic steatosis results from increased synthesis and substrate availability as well as reduced oxidation and export of fats in combination or alone. To define how single ethanol binge causes fat deposition in mice under the 2 dietary/nutritional conditions (ie, chow and Western diet), we measured hepatic levels of mRNAs for key genes in the pathways of *de novo* lipogenesis, fatty acid transport and oxidation, and intracellular lipid droplet lipolysis. As shown in Figure 3A, 1 dose of ethanol increased hepatic mRNA levels of several key lipogenic genes, including sterol-regulatory element-binding protein-1c (Srebp1c), carbohydrate response element-binding protein (Chrebp), acetyl CoA carboxylase 1 (Acc1), fatty acid synthase (Fasn), and stearoyl CoA desaturase-1 (Scd1) in mice on the chow diet. The western diet also increased hepatic expression of these lipogenic genes when compared with the chow diet. Binge ethanol feeding further augmented hepatic levels of mRNAs for the key *de novo* lipogenic genes Acc1, Fasn, and Scd1 in Western diet-fed mice. While Western diet feeding increased hepatic expression of the transcription factor peroxisome proliferator-activated receptor (PPARα) that governs fatty acid oxidation, a single binge of ethanol significantly suppressed PPARα mRNA expression in the liver regardless of diets. A similar pattern was observed for carnitine palmitoyltransferase-1A (Cpt1a), a target gene of PPARα. There were no significant differences among 4 groups in hepatic levels of mRNAs for the fatty acid transporter cluster determinant-36 (Cd36) and the intracellular lipolytic genes including adipose triglyceride lipase (Atgl), hormone-sensitive lipase (Hsl), and the lipolytic activator Comparative Gene Identification-58 (Cgi-58, also known as alpha/beta hydrolase domain-containing 5 or Abhd5).^19^

At the protein level (Figures 3B and C), single ethanol binge increased hepatic ACC and FASN in mice fed the chow but not the Western diet. As expected, mice fed a Western diet relative to those on the chow diet had a significant increase in hepatic ACC and FASN proteins. Consistent with mRNA changes, hepatic levels of CD36 and ATGL proteins remained unaltered among all groups. Different from CGI-58 mRNA expression, hepatic CGI-58 protein was elevated in mice fed Western diet relative to the chow diet or in ethanol-treated mice relative to those treated with the isocaloric dextrin-maltose solution. This was not surprising because CGI-58 is a lipid droplet-associated protein, and increased lipid droplets are expected to sequester more CGI-58 protein to their surfaces (Figure 2). Our findings at gene transcription and protein expression levels suggest that 1 binge of ethanol increases hepatic lipogenesis. Our data also suggest that single ethanol binge suppresses fatty acid oxidation similarly under both dietary conditions without altering fatty acid transport and intracellular lipid droplet lipolysis in the liver in mice.

### Western diet and single ethanol binge synergistically increase hepatic infiltration of neutrophils

**FIGURE 4:**
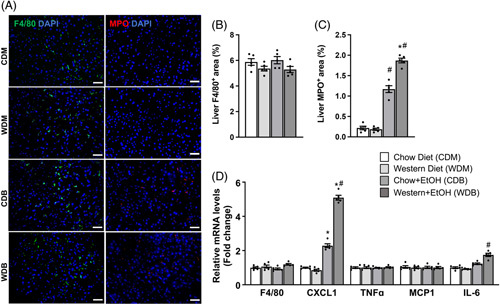
Western diet feeding and single ethanol binge synergistically increase hepatic neutrophils but not macrophages. The same mice described under Figure 1 were used. (A) Representative immunofluorescence images of the macrophage marker F4/80 and the neutrophil marker MPO in the liver. (B) Quantification of F4/80 and (**C**) MPO. (D) Hepatic mRNA levels of inflammatory genes include F4/80 (a macrophage marker) and Cxcl1 (a chemoattractant for neutrophils). Scale bar: 50 μm. *p < 0.05 (Western diet vs. chow diet); ^#^ *p* < 0.05 (binge ethanol vs. maltose solution).

A hallmark of ALD is the increased neutrophils in the liver.^20^ Interestingly, a single binge of ethanol sufficed to significantly increase the hepatic neutrophil number as evidenced by immunofluorescence staining of the neutrophil protein marker MPO as well as by qPCR analysis of the mRNA for C-X-C Motif Chemokine Ligand 1 (Cxcl1), a chemokine that is critically implicated in tissue neutrophil recruitment (Figure 4A-C). This increase was much greater in mice fed a Western diet than a chow diet, suggesting a synergistic effect of the Western diet plus ethanol binge. No alteration was observed among all groups in hepatic macrophage infiltration, as shown by immunofluorescence staining and qPCR analysis of the macrophage marker F4/80 (Figures 4A and C). Moreover, we did not observe significant alterations in hepatic levels of mRNAs for the tumor necrosis factor alpha (Tnfα) and monocyte chemoattractant protein-1 (Mcp1) among the 4 experimental groups, except IL-6 that was increased by binge ethanol feeding in Western diet-fed mice (Figure 4C). Our results collectively indicate that the Western diet exacerbates binge ethanol-induced hepatic infiltration of neutrophils.

### Western diet and single ethanol binge synergistically induce hepatic oxidative stress

**FIGURE 5:**
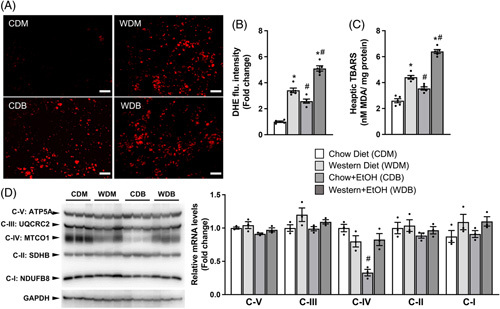
Western diet feeding and single ethanol binge synergistically induce hepatic oxidative stress. The same mice described under Figure 1 were used. (A) Representative images and quantification of Dihydroethidium (DHE) fluorescence signals of liver sections. (B) Hepatic contents of thiobarbituric acid reactive substances (TBARS) as (C) malondialdehyde (MDA). (D) Western blots and densitometry analysis of mitochondrial electron transport chain complex proteins in the liver. Scale bar: 100 μm. *p < 0.05 (Western diet vs. chow diet); ^#^ *p* < 0.05 (binge ethanol vs. maltose solution).

It has been well established that alcohol overconsumption causes hepatic oxidative stress.^21^ Single ethanol binge did not alter hepatic levels of mRNAs for ethanol-metabolizing enzymes, including alcohol dehydrogenase 1 (ADH1), aldehyde dehydrogenase 2 (ALDH2), and cytochrome p450 2E1 (Cyp2E1), in Western diet- or HFD-fed mice, though reducing ADH1 and Cyp2E1 in chow-fed mice (data not shown). Excessive fat accumulation may worsen oxidative stress caused by ethanol metabolism through lipotoxicity. Indeed, we found that a single ethanol binge resulted in a greater increase in hepatic reactive oxygen species (ROS) in mice fed the Western diet versus those on the chow diet, as revealed by staining of dihydroethidium (DHE), a fluorescence product that specifically reacts with intracellular superoxides (Figure 5A). Hepatic lipid peroxidation measured by malondialdehyde (MDA) production during thiobarbituric acid reactive substances (TBARS) assays was also the highest in mice fed Western diet plus ethanol binge (Figure 5B). Mitochondria are the major source of ROS. To determine whether the Western diet plus one ethanol binge caused any damage to mitochondrial oxidative phosphorylation, we measured hepatic levels of mitochondrial respiratory chain proteins. As shown in Figure 5C, we found that representative proteins of mitochondrial complexes I, II, III, and V were comparable among all groups. One binge of ethanol significantly reduced the complex IV component MTCO1 in mice fed the chow but not the Western diet. These findings suggest that exacerbation of ethanol-induced hepatic oxidative stress in Western diet-fed mice may not result from mitochondrial dysfunction.

### Western diet and single ethanol binge synergistically induce hepatic endoplasmic reticulum (ER) stress

**FIGURE 6:**
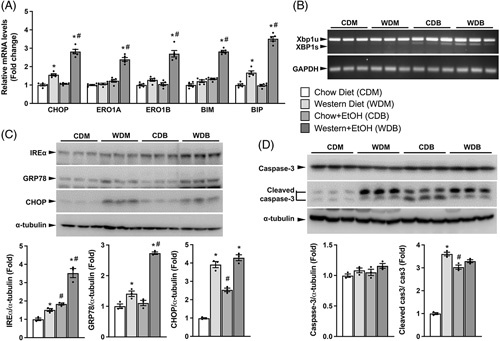
Western diet plus single ethanol binge synergistically induces hepatic ER stress. The same mice described under Figure 1 were used. (A) Relative hepatic levels of mRNA for UPR-related genes. (B) Reverse transcriptase PCR assay of Xbp1 splicing using an equal amount of hepatic total RNAs from each sample. (**C**) Western blots and densitometry analysis of hepatic levels of IREα, GRP78, and CHOP proteins. (D) Western blots and densitometry analysis of hepatic levels of caspase 3 and cleaved caspase 3 proteins. *p < 0.05 (Western diet vs. chow diet); ^#^ *p* < 0.05 (binge ethanol vs. maltose solution).

The unfolded protein response (UPR) pathway is an adaptive mechanism by which cells prevent overload of unfolded proteins in the ER.^22^ Dysregulation of UPR may result in ER stress, which has been implicated in the pathogenesis of many diseases, including alcoholic and non-alcoholic liver diseases.^23–25^ We hypothesized that a combination of the Western diet and binge ethanol may exacerbate hepatic ER stress, thereby worsening liver pathologies induced by the Western diet or ethanol alone. To test this hypothesis, we examined hepatic mRNA and protein expression levels of key genes in the UPR signal transduction pathway. Consistent with our hypothesis, hepatic mRNA levels of CCAAT-enhancer-binding protein homologous protein (CHOP) and the 78-kDa glucose-regulated protein (GRP78, also known as binding immunoglobulin protein or BIP) exhibited a significant increase in Western diet-fed mice relative to chow-fed mice while those of endoplasmic reticulum oxidoreductin-1a (ERO1a), ERO1b, and Bcl-2-interacting mediator of cell death (BIM) remained unaltered between the two dietary groups (Figure 6A). One binge of ethanol raised hepatic levels of mRNAs for all the genes mentioned above in mice fed Western diet but not in those on the chow diet. Ethanol and the Western diet clearly exerted a synergistic effect on the transcriptional upregulation of these UPR genes in the liver. The activity of inositol-requiring transmembrane kinase/endoribonuclease 1 (IRE-1) assessed by X-Box binding protein-1 (Xbp1) splicing was largely unchanged between Western diet- and chow diet-fed mice, but significantly increased by ethanol binge regardless of diets (Figure 6B). Hepatic levels of IRE1α, BIP/GRP78, and CHOP proteins were also significantly elevated in mice fed Western diet versus chow diet (Figure 6C). Ethanol binge increased hepatic IRE1α and CHOP but not GRP78/BIP proteins in chow-fed mice. A synergistic effect was observed for hepatic IRE1α and BIP/GRP78 protein levels in mice subjected to both the Western diet and ethanol, though CHOP protein did not further increase. These results indicate that the Western diet augments ethanol binge-induced ER stress.

Since ER stress induces apoptosis in the liver,^26^ we examined whether the ER stress caused by our dietary regimens was associated with alterations in this cellular process. We found that the Western diet substantially increased hepatic cleaved caspase 3, a protein that mediates apoptosis (Figure 6D). Binge ethanol also increased cleaved caspase 3 protein in the liver in mice on the chow diet, but it did not further augment Western diet-induced caspase 3 cleavage. The results from comparative studies of ER stress and caspase 3 cleavage suggest a positive association between ER stress and apoptosis in our animals.

## DISCUSSION

With frequent coexistence of ALD and NAFLD, overnutrition has been recognized as a major risk factor for both diseases.^27^ Increases in advanced ALD in young people have resulted in more research on alcohol binge-induced liver injury because this age group has a high prevalence of binge drinking.^4–8^ Realizing these 2 facts, scientists have started to study the interactions of ethanol binge and overnutrition. It was reported that the single ethanol binge caused more severe liver injury in mice fed an HFD for 3 days or 3 months compared to those on the chow diet.^28^ However, HFDs contain a very low amount (~0.015%, w/w) of cholesterol and 60% energy as fats. Despite the wide use of HFD in obesity research, low-cholesterol HFDs are rarely consumed in Western and developed societies. The widely consumed diets in these societies are Western diets, typically containing ~ 40% energy from fats and ~0.2% (w/w) of cholesterol. In this study, we established within 3 weeks a novel murine model of acute liver injury by mimicking human diets and alcohol drinking behaviors in developed countries. The liver injury, as evidenced by elevated serum ALT and AST activities, was much more severe in this Western diet plus single ethanol binge model than Western diet or ethanol binge alone. The model also displays robust hepatic steatosis, neutrophil infiltration, oxidative stress, and ER stress. The establishment of our animal model is rapid and simple, requiring no special cages or equipment. This animal model is highly relevant to human lifestyles and behaviors and is a convenient tool for the definition of how binge drinking and Western diets interact to synergistically induce acute liver injury and steatohepatitis. In addition, since binge drinking and chronic alcohol overconsumption cause liver injury likely by similar mechanisms and repeated alcohol binges as additional hits may worsen pre-existing liver pathologies leading to disease progression,^29^ further mechanistic studies of our animal model and its modified versions (eg, including multiple ethanol binges and/or chronic ethanol feeding) may lead to novel strategies for the management of chronic ALD and NAFLD, the two most prevalent liver diseases in Western countries.

We chose three weeks of Western diet feeding prior to the ethanol binge because this dietary regimen was sufficient to elevate baseline levels of serum AST and ALT activities without adversely affecting the general health. In the literature, various dietary durations were employed in studies of cholesterol-containing diet-induced hepatic steatosis and liver injury. One study fed mice a high-cholesterol diet for up to 14 months^30^ while another for 1 week,^31^ both of which showed increased AST and ALT. There were many studies using the dietary durations in between, and the diets containing ~0.2% (w/w) cholesterol was considered to be most relevant to human diets and used in most animal research of NAFLD/NASH.^32,33^

The progression of ALD, like NAFLD, may involve multiple hits.^13^ The first hit of ALD may not be alcohol but the liver injury induced by nutritional, environmental, or genetic factors, including chronic consumption of cholesterol-containing Western diets.^27^ Krishnasamy et al^34^ reported that mice fed 3 months of ethanol plus cholesterol (0.5%, w/v)-containing Lieber-DeCarli liquid diet developed severe steatohepatitis and early liver fibrosis without altering hepatic protein expression of the ethanol metabolic enzymes ADH1, Cyp2E1, and ALDH2. In the present study, ethanol binge did not change hepatic levels of mRNAs for these proteins in Western diet or HFD-fed mice (data not shown). Schonfeld et al showed that mice fed a Western diet similar to ours in combination with chronic alcohol consumption in drinking water for 16 weeks exhibited more severe steatosis, inflammation, and fibrosis in the liver compared to control mice fed either Western diet alone for 16 weeks or Lieber-DeCarli liquid diet alone for 3 weeks.^35,36^ A key feature of the Western diet is its high-cholesterol content. After the ethanol binge, Western diet-fed mice had the highest hepatic content of free cholesterol and serum activities of AST and ALT. Excess cholesterol is known to be cytotoxic.^15^ The positive association between hepatic cholesterol content and serum AST/ALT activities may imply a potential role of cholesterol in mediating ethanol-induced liver injury, though their causal relationship has yet to be established. Nonetheless, our findings in this study suggest that moderate liver injury induced by 3 weeks of Western diet feeding is sufficient for a single dose of ethanol to cause severe liver injury and steatosis in mice. Multiple bouts of binge drinking may tip the balance of injury and repair, leading to more severe inflammatory responses and ultimately scaring of the liver, which may explain why liver cirrhosis-related mortality was increased in young people.^4^

In our study, Western diet feeding for three weeks resulted in a significant increase in serum concentrations of NEFAs, and single dose of ethanol exacerbated this increase (Figure 2). This finding was not surprising because Western versus chow diets have higher amounts of fats and ethanol feeding has been known to stimulate adipose lipolysis to release fatty acids to bloodstream.^37^ It was reported that about 60% of liver fatty acids are derived from adipose tissues.^18^ Fatty acids are ligands for PPAR transcription factors.^38^ When more fatty acids enter the liver from blood, one would expect activation of PPARs and upregulation of PPAR target genes. Consistently, hepatic levels of mRNAs for the fatty acid uptake transporter Cd36, a PPAR-gamma target,^39^ and for the fatty acid oxidation enzyme Cpt1a, a PPAR-alpha target^40^ were upregulated by Western diet feeding. The upregulation of hepatic fatty acid oxidation may be a protective mechanism, preventing excessive overaccumulation of fats when dealing with excess fatty acids.

Alcohol intake is known to suppress the hepatic transcription of genes related to fatty acid oxidation.^41^ Indeed, ethanol binge in the present study caused a significant reduction in hepatic mRNA levels of Ppar-α and Cpt1a. Interestingly, acute ethanol binge completely blocked the Western diet-associated upregulation of Ppar-α and Cpt1a mRNAs, which, together with high concentrations of circulating fatty acids, may underlie the exacerbation of hepatic steatosis in our Western diet plus ethanol binge animals. Lipogenesis may not contribute much to this exacerbation because acute ethanol binge increased hepatic expression of lipogenic genes to a similar level in both chow and Western diet groups (Figure 3). In addition, cytosolic lipid droplet lipolysis may have a limited role in aggravating hepatic steatosis in our animal model because hepatic expression of key lipolytic genes HSL, ATGL, and CGI-58 remained unchanged between the mice fed Western diet alone and those fed Western diet plus ethanol binge.

A hallmark of ALD is hepatic oxidative stress.^21^ Consistent with this established role, binge ethanol feeding caused hepatic oxidative stress in our chow-fed mice. Western diet alone also increased oxidative stress, and this increase was further exacerbated by acute ethanol binge in our model. Mitochondria are the major source of reactive oxygen species.^42^ However, the oxidative stress in our model may not be attributable to mitochondrial dysfunction because no changes in hepatic expression of key proteins in the 5 mitochondrial oxidative phosphorylation complexes were observed (Figure 5). The most likely cause appears to be fat overaccumulation and associated lipotoxicity and lipid peroxidation.

Western diet plus binge ethanol feeding synergistically increased hepatic infiltration of neutrophils, which was associated with a selective and synergistic increase in Cxcl1 mRNA expression in the liver (Figure 4). Western diet *per se* did not alter hepatic Cxcl1 mRNA expression and neutrophils, indicating a dietary cholesterol-independent process. Cxcl1 transcription and neutrophil infiltration in the liver were largely ethanol dependent, consistent with the notion that neutrophil infiltration is a hallmark of ALD.^20^

It has been well-recognized that ER stress can be the cause and result of NAFLD and ALD.^43–45^ In our study, single dose of ethanol aggravated Western diet-induced hepatic ER stress, suggesting a synergistic effect of Western diet and ethanol binge on ER stress. An intriguing finding in the present study was that Western diet feeding for 3 weeks markedly enhanced the cleavage of hepatic caspase 3. Caspase 3 cleavage promotes apoptosis and inflammation. Our finding thus indicates that Western diets are pro-apoptotic and pro-inflammatory, at least in mice. Acute ethanol binge did not aggravate Western diet-associated caspase 3 cleavage. Considering the synergistic effect of the Western diet plus ethanol binge on hepatic Cxcl1 expression and neutrophil infiltration, the lack of synergistic caspase 3 cleavage suggests a caspase 3 cleavage-independent mechanism or mechanisms that are downstream of caspase 3 cleavage with the cleavage as a permissive factor for exacerbating alcohol-induced Cxcl1 expression and neutrophil infiltration.

In conclusion, we established within 3 weeks a novel murine model of acute liver injury by mimicking human diets and alcohol drinking behaviors in developed societies. This Western diet plus binge ethanol feeding model recapitulates many features of liver pathologies observed in those with the coexistence of ALD and NAFLD in Western communities. Further studies and modifications of this simple animal model may lead to novel preventive and therapeutic strategies for both ALD and NAFLD.

## Supplementary Material

## References

[1] Eslam M Sanyal AJ George J. International Consensus P. MAFLD: A Consensus-driven proposed nomenclature for metabolic associated fatty liver disease. Gastroenterology. 2020;158:1999–2014. e1991. 32044314 10.1053/j.gastro.2019.11.312

[2] Sharma P Arora A. Clinical presentation of alcoholic liver disease and non-alcoholic fatty liver disease: spectrum and diagnosis. Transl Gastroenterol Hepatol. 2020;5:19. 32258523 10.21037/tgh.2019.10.02 PMC7063523

[3] Ventura-Cots M Watts AE Bataller R. Binge drinking as a risk factor for advanced alcoholic liver disease. Liver Inter. 2017;37:1281; official journal of the International Association for the Study of the Liver. 10.1111/liv.13482 PMC5656398 28845617

[4] Tapper EB Parikh ND. Mortality due to cirrhosis and liver cancer in the United States, 1999-2016: observational study. BMJ. 2018;362:k2817. 30021785 10.1136/bmj.k2817 PMC6050518

[5] Kanny D Naimi TS Liu Y. Annual total binge drinks consumed by U.S. Adults, 2015. Am J Prev Med. 2018;54:486–496. 29555021 10.1016/j.amepre.2017.12.021 PMC6075714

[6] Akseer N Al-Gashm S Mehta S. Global and regional trends in the nutritional status of young people: a critical and neglected age group. Ann N Y Acad Sci. 2017;1393:3–20. 28436100 10.1111/nyas.13336

[7] Gonzales KR Largo TW Miller C. Consumption of alcoholic beverages and liquor consumption by Michigan High School Students, 2011. Prev Chronic Dis. 2015;12:E194. 26564010 10.5888/pcd12.150290 PMC4651140

[8] Dawson DA Goldstein RB Saha TD. Changes in alcohol consumption: United States, 2001-2002 to 2012-2013. Drug Alcohol Depend. 2015;148:56–61. 25620731 10.1016/j.drugalcdep.2014.12.016 PMC4330106

[9] Wegner SA Pollard KA Kharazia V. Limited excessive voluntary alcohol drinking leads to liver dysfunction in mice. Alcohol Clin Exp Res. 2017;41:345–358. 28103636 10.1111/acer.13303 PMC5636002

[10] Ruhl CE Everhart JE. Joint effects of body weight and alcohol on elevated serum alanine aminotransferase in the United States population. Clin Gastroenterol Hepatol. 2005;3:1260–1268. 16361053 10.1016/s1542-3565(05)00743-3

[11] Lau K Baumeister SE Lieb W. The combined effects of alcohol consumption and body mass index on hepatic steatosis in a general population sample of European men and women. Aliment Pharmacol Ther. 2015;41:467–476. 25588768 10.1111/apt.13067

[12] Inan-Eroglu E Huang BH Ahmadi MN. Joint associations of adiposity and alcohol consumption with liver disease-related morbidity and mortality risk: findings from the UK Biobank. Eur J Clin Nutr. 2022;76:74–83. 34059777 10.1038/s41430-021-00923-4

[13] Dasarathy S Brown JM. Alcoholic liver disease on the rise: Interorgan cross talk driving liver injury. Alcohol Clin Exp Res. 2017;41:880–882. 28295407 10.1111/acer.13370 PMC5405002

[14] Buettner R Scholmerich J Bollheimer LC. High-fat diets: modeling the metabolic disorders of human obesity in rodents. Obesity (Silver Spring). 2007;15:798–808. 17426312 10.1038/oby.2007.608

[15] Song Y Liu J Zhao K. Cholesterol-induced toxicity: An integrated view of the role of cholesterol in multiple diseases. Cell Metab. 2021;33:1911–1925. 34562355 10.1016/j.cmet.2021.09.001

[16] Yang P Wang Y Tang W. Western diet induces severe nonalcoholic steatohepatitis, ductular reaction, and hepatic fibrosis in liver CGI-58 knockout mice. Sci Rep. 2020;10:4701. 32170127 10.1038/s41598-020-61473-6 PMC7070035

[17] Zhong W Zhao Y Tang Y. Chronic alcohol exposure stimulates adipose tissue lipolysis in mice: role of reverse triglyceride transport in the pathogenesis of alcoholic steatosis. Am J Pathol. 2012;180:998–1007. 22234172 10.1016/j.ajpath.2011.11.017 PMC3349880

[18] Donnelly KL Smith CI Schwarzenberg SJ. Sources of fatty acids stored in liver and secreted via lipoproteins in patients with nonalcoholic fatty liver disease. J Clin Invest. 2005;115:1343–1351. 15864352 10.1172/JCI23621 PMC1087172

[19] Yu L Li Y Grisé A. CGI-58: versatile regulator of intracellular lipid droplet homeostasis. Adv Exp Med Biol. 2020;1276:197–222. 32705602 10.1007/978-981-15-6082-8_13 PMC8063591

[20] Tang J Yan Z Feng Q. The Roles of Neutrophils in the Pathogenesis of Liver Diseases. Front Immunol. 2021;12:625472. 33763069 10.3389/fimmu.2021.625472 PMC7982672

[21] Yang YM Cho YE Hwang S. Crosstalk between oxidative stress and inflammatory liver injury in the pathogenesis of alcoholic liver disease. Int J Mol Sci. 2022;23:774. 35054960 10.3390/ijms23020774 PMC8775426

[22] Lindholm D Korhonen L Eriksson O. Recent insights into the role of unfolded protein response in ER stress in health and disease. Front Cell Dev Biol. 2017;5:48. 28540288 10.3389/fcell.2017.00048 PMC5423914

[23] Rutkowski DT Wu J Back SH. UPR pathways combine to prevent hepatic steatosis caused by ER stress-mediated suppression of transcriptional master regulators. Dev Cell. 2008;15:829–840. 19081072 10.1016/j.devcel.2008.10.015 PMC2923556

[24] Lebeaupin C Vallee D Hazari Y. Endoplasmic reticulum stress signalling and the pathogenesis of non-alcoholic fatty liver disease. J Hepatol. 2018;69:927–947. 29940269 10.1016/j.jhep.2018.06.008

[25] Song Q Chen Y Wang J. ER stress-induced upregulation of NNMT contributes to alcohol-related fatty liver development. J Hepatol. 2020;73:783–793. 32389809 10.1016/j.jhep.2020.04.038 PMC8301603

[26] Lan J Zhong Z Wang Y. Endoplasmic reticulum stress induces liver cells apoptosis after brain death by suppressing the phosphorylation of protein phosphatase 2A. Mol Med Rep. 2020;21:567–574. 31974600 10.3892/mmr.2019.10874 PMC6947944

[27] Skinner RC Hagaman JA. The interplay of Western diet and binge drinking on the onset, progression, and outlook of liver disease. Nutr Rev. 2022;80:503–512. 33969426 10.1093/nutrit/nuab031

[28] Chang B Xu MJ Zhou Z. Short- or long-term high-fat diet feeding plus acute ethanol binge synergistically induce acute liver injury in mice: an important role for CXCL1. Hepatology. 2015;62:1070–1085. 26033752 10.1002/hep.27921 PMC4589443

[29] Massey VL Arteel GE. Acute alcohol-induced liver injury. Front Physiol. 2012;3:193. 22701432 10.3389/fphys.2012.00193 PMC3372892

[30] Zhang X Coker OO Chu ES. Dietary cholesterol drives fatty liver-associated liver cancer by modulating gut microbiota and metabolites. Gut. 2021;70:761–774. 32694178 10.1136/gutjnl-2019-319664 PMC7948195

[31] Castellanos-Tapia L Tejero-Barrera ME Salas-Silva S. Mediterranean-like mix of fatty acids induces cellular protection on lipid-overloaded hepatocytes from western diet fed mice. Ann Hepatol. 2020;19:489–496. 32663612 10.1016/j.aohep.2020.06.005

[32] Farrell G Schattenberg JM Leclercq I. Mouse models of nonalcoholic steatohepatitis: toward optimization of their relevance to human nonalcoholic steatohepatitis. Hepatology. 2019;69:2241–2257. 30372785 10.1002/hep.30333

[33] Charlton M Krishnan A Viker K. Fast food diet mouse: novel small animal model of NASH with ballooning, progressive fibrosis, and high physiological fidelity to the human condition. Am J Physiol Gastrointest Liver Physiol. 2011;301:G825–G834. 21836057 10.1152/ajpgi.00145.2011 PMC3220319

[34] Krishnasamy Y Ramshesh VK Gooz M. Ethanol and high cholesterol diet causes severe steatohepatitis and early liver fibrosis in mice. PLoS One. 2016;11:e0163342. 27676640 10.1371/journal.pone.0163342 PMC5038945

[35] Schonfeld M Averilla J Gunewardena S. Male-specific activation of lysine demethylases 5B and 5C Mediates Alcohol-Induced Liver Injury and Hepatocyte Dedifferentiation. Hepatol Commun. 2022;6:1373–1391. 35084807 10.1002/hep4.1895 PMC9134811

[36] Schonfeld M O’Neil M Villar MT. A Western diet with alcohol in drinking water recapitulates features of alcohol-associated liver disease in mice. Alcohol Clin Exp Res. 2021;45:1980–1993. 34523155 10.1111/acer.14700 PMC9006178

[37] Wei X Shi X Zhong W. Chronic alcohol exposure disturbs lipid homeostasis at the adipose tissue-liver axis in mice: analysis of triacylglycerols using high-resolution mass spectrometry in combination with in vivo metabolite deuterium labeling. PLoS One. 2013;8:e55382. 23405143 10.1371/journal.pone.0055382 PMC3566154

[38] Varga T Czimmerer Z Nagy L. PPARs are a unique set of fatty acid regulated transcription factors controlling both lipid metabolism and inflammation. Biochim Biophys Acta. 2011;1812:1007–1022. 21382489 10.1016/j.bbadis.2011.02.014 PMC3117990

[39] Zhu H Jia Z Misra H. Oxidative stress and redox signaling mechanisms of alcoholic liver disease: updated experimental and clinical evidence. J Dig Dis. 2012;13:133–142. 22356308 10.1111/j.1751-2980.2011.00569.x PMC3297983

[40] Rakhshandehroo M Knoch B Muller M. Peroxisome proliferator-activated receptor alpha target genes. PPAR Res. 2010;2010:612089. 20936127 10.1155/2010/612089 PMC2948931

[41] Jeon S Carr R. Alcohol effects on hepatic lipid metabolism. J Lipid Res. 2020;61:470–479. 32029510 10.1194/jlr.R119000547 PMC7112138

[42] Andreyev AY Kushnareva YE Starkov AA. Mitochondrial metabolism of reactive oxygen species. Biochemistry (Mosc). 2005;70:200–214. 15807660 10.1007/s10541-005-0102-7

[43] Maiers JL Malhi H. Endoplasmic reticulum stress in metabolic liver diseases and hepatic fibrosis. Semin Liver Dis. 2019;39:235–248. 30912096 10.1055/s-0039-1681032 PMC6530577

[44] Liu X Green RM. Endoplasmic reticulum stress and liver diseases. Liver research. 2019;3:55–64. 32670671 10.1016/j.livres.2019.01.002 PMC7363397

[45] Han J Kaufman RJ. The role of ER stress in lipid metabolism and lipotoxicity. J Lipid Res. 2016;57:1329–1338. 27146479 10.1194/jlr.R067595 PMC4959874

