# Supplementary material for: Single ethanol binge causes severe liver injury in mice fed Western diet
Source: Hepatol Commun. 2023 Jun 14;7(7):e00174. doi: 10.1097/HC9.0000000000000174 (PMC10270551; doi:10.1097/HC9.0000000000000174)
Supplement: Supplementary file 1 [file hc9-7-e00174-s001.docx]

**Supplementary Table 1.** The primers used for qPCR in this study

| Primer | Forward (5’ to 3’) | Reverse (5’ to 3’) |
| --- | --- | --- |
| Acc1 | TGACAGACTGATCGCAGAGAAAG | TGGAGAGCCCCACACACA |
| Atgl | CAACGCCACTCACATCTACGG | GGACACCTCAATAATGTTGGCAC |
| Bim | GCTCCTGTGCAATCCGTATC | GCCCCTACCTCCCTACAGAC |
| Bip | GAAAGGATGGTTAATGATGCTGAG | GTCTTCAATGTCCGCATCCTG |
| Cd36 | TGCACCACATATCTACCAAA | TTGTAACCCCACAAGAGTTC |
| Cgi-58 | ATCTTTGGAGCCCGATCCT | CTTCTGGCTGATCTGCATACAC |
| Chop | GACCAGGTTCTGCTTTCAGG | CAGCGACAGAGCCAGAATAA |
| Cheep | CCTCACTTCACTGTGCCTCA | ACAGGGGTTGTTGTCTCTGG |
| Cpt1a | CACCAACGGGCTCATCTTCTA | CAAAATGACCTAGCCTTCTATCGAA |
| Cxcl1 | TCCCCAAGTAACGGAGAAAGAA | TGTCAGAAGCCAGCGTTCAC |
| Cyp7a1 | AGCAACTAAACAACCTGCCAGTACTA | GTCCGGATATTCAAGGATGCA |
| Cyp8b1 | GCCTTCAAGTATGATCGGTTCCT | GATCTTCTTGCCCGACTTGTAGA |
| Cyp27a1 | GGAGGGCAAGTACCCAATAAGA | TGCGATGAAGATCCCATAGGT |
| Ero1a | CACAGGTACAGTCGTCCAGGT | CTTGCTCGTTGGACTCCTG |
| Ero1b | TGACAAAAAGGGGGCCAAGT | TATCGCACCCAACACAGTCC |
| F4/80 | CTTTGGCTATGGGCTTCCAGTC | GCAAGGAGGACAGAGTTTATCGTG |
| Fasn | GCTGCGGAAACTTCAGGAAAT | AGAGACGTGTCACTCCTGGACTT |
| Hsl | GCTGGGCTGTCAAGCACTGT | GTAACTGGGTAGGCTGCCAT |
| Il-1b | CAACCAACAAGTGATATTCTCC | GATCCACACTCTCCAGCTGCA |
| Il-6 | TCCATCCAGTTGCCTTCTTG | TTCCACGATTTCCCAGAGAAC |
| Mcp1 | CCTGGATCGGAACCAAATGA | AGACCTTAGGGCAGATGCAGTT |
| Pparα | ACAAGGCCTCAGGGTACCA | GCCGAAAGAAGCCCTTACAG |
| Scd1 | TTCTCAGAAACACACGCCGA | AGCTTCTCGGCTTTCAGGTC |
| Srebp-1c | GGAGCCATGGATTGCACATT | GGCCCGGGAAGTCACTGT |
| Tnfα | CTGAGGTCAATCTGCCCAAGTAC | CTTCACAGAGCAATGACTCCAAAG |
